# Pseudo “insulin allergy”

**DOI:** 10.4103/0973-3930.45274

**Published:** 2008

**Authors:** Pradeep Raman Chettiar, Nishanth Sanalkumar, Mathew John

**Affiliations:** Department of Endocrinology and Diabetes, Kerala Institute of Medical Sciences, Trivandrum - 695 011, India

**Keywords:** Abscess, allergy, insulin

## Abstract

Allergy to human insulin is relatively rare in clinical practice. This report describes a patient referred for suspected “insulin allergy” due to lesions appearing at all sites of insulin injection. Careful evaluation confirmed contamination of the insulin syringes due to faulty techniques used by the patient. The report discusses the various types of insulin allergies and the need for proper diabetic education to avoid such infections.

## Introduction

Insulin allergy in patients with diabetes mellitus on insulin treatment is a rare condition.[1] Adverse reactions to insulin have decreased significantly since the introduction of human insulin.[1] True allergies to insulin being rare; other closely related conditions should be suspected when patients present with suspected insulin allergy.

## Case Report

A 76-year-old male was referred to endocrine services for suspected “insulin allergy.” He was diagnosed to have diabetes mellitus 8 years back and was on treatment with oral hypoglycemics till 6 months back. He was admitted with malignant otitis externa following which he was started on premixed human insulin (30/70) for uncontrolled blood sugars. During his hospitalization for 7 days, he was on treatment with basal bolus insulin with regular and NPH human insulin. There were no adverse skin reactions to insulin during the hospital stay. He gives a history of left middle cerebral artery infarct 6 years back, following which he is on treatment with T. Clopidogrel. He was also on T. Losartan 50 mg daily for essential hypertension and T. Atorvastatin 10 mg daily for LDL predominant dyslipidemia. He was never known to have any drug or food allergies. He does not give any family history of allergic or autoimmune diseases.

Since hospital discharge, the patient complains of multiple lesions appearing on the site of insulin injection. It starts as a pruritic weal, which becomes an abscess and later breaks down to form an ulcer. On examination, there were multiple abscesses scattered over the anterior and lateral surfaces of thighs, arms and forearms. Most of the abscesses were in various degrees of healing. Clinical examination confirmed residual right hemiparesis, peripheral neuropathy, and mild nonproliferative diabetic retinopathy.

Biochemical investigations: hemoglobin: 12 gm/dl; total WBC count: 12,300 /mm^3^ P 72%, L 24%, E 4%; S. Creatinine: 0.9 mg/dl; HbA1c: 9.5%; FPG: 188 mg/dl and PPG 289 mg/dl. Pus culture from one of the abscess over the thigh yielded methicillin-sensitive *Staphylococcus aureus*.

Since the patient did not have any allergic reactions to insulin during hospitalization, problems associated with insulin delivery or contamination was suspected. The patient and his daughter were queried about various aspects of insulin delivery. Insulin was delivered by 40 U/ml syringes, which were disposed after two injections. The technique of injections, storage of insulin and cleaning of skin before injections were confirmed. On careful questioning, the daughter reported that they flushed the syringes with warm water and wiped the needles dry before injecting. He was treated with oral Cloxacillin and was advised to shift the injection site to abdomen. He was instructed to use the syringes only once and never to clean or touch the needles. Follow up of the patient revealed no further abscess formation

## Discussion

Although the prevalence of suspected insulin allergy have been reported as high as 2.5%, diagnosis should be more accurate since less than one-third of patients are finally diagnosed to have true insulin allergy.[2] Although true insulin allergy is rare, allergic reaction to various components of the insulin preparations is more common.[1] Allergic reactions to insulin include immediate type IgE-mediated reactions, type 3 immune complex type (Arthus reaction – localized or serum sickness –generalized) or delayed type hypersensitivity reactions.[1] Methods used in literature to deal with human insulin allergy are to change to analogs like Aspart or Lispro, continuous subcutaneous insulin infusion and desensitization with progressively increasing doses of subcutaneous or intravenous insulin.[1,2]

In a series of 22 patients with suspected insulin allergy, poor injection technique (*n* = 5) and skin diseases (*n* = 3) contributed to the suspected allergy.[3] Insulin injection abscesses occur in patients with diabetes and are mainly due to *Staphylococcus aureus*.[4] Unusual organisms include atypical mycobacteria and Streptococci.[4] Perinephric abscess has been reported due insulin syringe reuse in a patient with diabetes mellitus.[5]

In our patient, poor insulin technique due to improper diabetic education lead to injection abscesses. Evidence from literature shows that diabetic education results in improved metabolic control, reduced risk of acute and chronic diabetic complications, reduced cost of treatment and reduced hospital admissions.[6] There is a scarcity of trained diabetic educators in India.[7] Diabetic education focusing on insulin administration would have helped our patient prevent this episode.
